# Based on Role Expectation to Discuss Role Ambiguity and Practice of University Teachers in Business Administration

**DOI:** 10.3389/fpsyg.2022.789806

**Published:** 2022-11-21

**Authors:** Yun Deng, Haimei Zeng, Anxin Xu, Youcheng Chen

**Affiliations:** ^1^College of Business Administration, Fujian Business University, Fuzhou, China; ^2^Anxi College of Tea Science, Fujian Agriculture and Forestry University, Quanzhou, China; ^3^College of Management and Economics, Fujian Agriculture and Forestry University, Fuzhou, China

**Keywords:** university teachers in business administration, role ambiguity, role expectation, role practice, legitimacy

## Abstract

As a result of social change, the issues, such as the complexity of family structure and increasing student problems, are becoming more complicated. Both schools and parents have high expectations of teachers and expect them to solve the problems. Considering the many different factors involved in this issue, this could lead to a psychological and physiological imbalance in teachers, especially in relation to their emotions which results in role ambiguity. The participants of this study consisted of teachers in business administration departments of universities in China. During the data collection, 450 copies of the questionnaire were distributed. A total of 363 valid copies were retrieved, with a retrieval rate of 81%. It can be concluded that the results of this study can help national university faculty present professional spirit and attitude toward the professional service, which in turn promotes the standard of national education in universities.

## Introduction

Education has improved greatly in recent years. Likewise, parents are gradually placing more emphasis on the importance of their children’s education, especially on the quality of teaching. Under the impact of the low birth rate, many parents are concerned about education. At the same time, concerns about teacher competence are worth discussing. Regardless of the high investment in education, access to good software–hardware in schools, and higher quality students, these components are meaningless without a “good teacher.” Teachers play an important role in the effectiveness of education. In recent years, many serious crimes have caused the society to worry about the shortage of manpower and insufficient expertise or skills on campus. Since school work is done by general teachers, counseling work cannot be done effectively. Prior to the outbreak of the incidents, education staffs are unaware of the importance of counseling and ignore the hidden signs of the crisis on campus, for example, bullying, gender equity, and high-risk students. Student-related problems can be ignored and may not be seen as a problem for the future. In addition, many people in the society do not have a proper understanding of school work, have false expectations of teachers’ work, and falsely attribute student responsibility to teachers, believing that teachers can and should do everything. Such a strong role pressure causes deep frustration and helplessness among teachers, who gradually lose their passion for the work. The reasons behind this are the teachers playing complicated and multiple roles. They are responsible for guiding students with deviant behaviors, consulting with teachers or parents, and helping with work-related issues in schools.

This leads to excessive pressure and burnout among teachers and even makes them consider changing jobs. Teachers start to procrastinate and get tired. They not only have to think about problems, but also control their emotions. They also need to improve themselves and find a way to take care of themselves after work. Modern national education reform is about promoting the performance and quality of national education. National education reform is a recent trend, but its effectiveness depends on the front-line executors, that is, the cooperation of teachers. Without a “good” teacher, the large investment in education, the good software–hardware of schools, and the high quality of students would be “in vain.” It is as in the saying “excellent teachers build a nation,” which applies to the past and also to the present and to education at the national level and abroad. Education, as a fundamental task that is crucial for generations, influences the future development and competitiveness of a nation. Teachers in the new age should present Pädagogishe Liebe (pedagogical love), professional competence, and capacity for the future. They should also stick to student-centeredness in classrooms, diligently and orderly develop the imperceptible and mind-changing educational function, and bear the burden of educational effectiveness. It can be seen that teachers’ classroom practices emphasize the promotion of educational performance and quality. Obviously, the effectiveness of teachers’ role practices is the key to the success of education reform; the importance of teachers’ role practices in educational effectiveness is obvious. From the perspective of school education, teacher’s performance can directly affect the effectiveness of the school. In other words, teacher’s performance directly affects the achievement of schools’ educational goals and is indirectly related to the students’ attitudes and expectations for learning performance.

The profession of teaching requires a high level of expertise, so educators should be treated as professionals and teachers’ specialization should be confirmed to promote the teachers’ professional development. The increased level of knowledge in recent years has made parents more concerned about their children’s learning equity. This may lead to the differences between parents and teachers on the essence of teaching, learning, and evaluation, but the conflict is often heard. In this case, curriculum, instruction, and performance contents are often affected by the parents, which prevents teachers from teaching with professional knowledge–competence and developing professional autonomy. In addition, due to the social change, the issues, such as the complexity of family structure and the increasing student problems, are becoming more complicated. Accordingly, schools sometimes have to take over the function of the family. Teachers are on the front-line people and experience bridging families and schools most of the time.

The surprise attack of COVID-19 has led to a radical change in the world order. The countermeasures are proclaimed in the framework of the state of emergency. School education with the face-to-face teaching environment bears the brunt of campus closure and cease of teaching and learning activities. However, many schools have switched to distance education in order not to interrupt learning (Kamali, 2021). Distance education practiced due to COVID-19 can be called “emergent distance teaching,” because teachers and students were in a hurry to implement and adapt to the changes within a short period of time. Many schools were not prepared well, and teachers were not familiar with distance education technologies and teaching methods. The process of improving distance education and increasing learning effectiveness is challenging (Marinoni et al., 2020). Teachers must adapt teaching models that are completely different from those of the past. These new teaching models were complicated and integrated with various advanced technologies. Since teachers did not experience such a situation before, nor had the necessary training, some teachers seemed to suffer from teaching frustration and psychological stress (Sadeghi, 2019).

Both the schools and parents have high expectations of teachers and expect them to solve the problems. Considering the many different factors involved in this issue, this could lead to a psychological and physiological imbalance in teachers, especially in relation to their emotions which results in role ambiguity. The review of the literature on teacher role and work revealed that there exist many studies that address role ambiguity, role expectation, job satisfaction, and job pressure (Lin and Ling, 2018; Amiruddin, 2019; Antwi et al., 2019; Wang et al., 2020). However, it was found that research on role ambiguity, role expectation, and practice is scarce, and research findings are mixed. The existing research on role ambiguity and role expectation mainly focused on the health-care workers, social workers, associate counselors, and human resource professionals (Mañas et al., 2018; Kokoroko and Sanda, 2019; Maden-Eyiusta, 2019), but the studies rarely focused on the university faculty. The research on professionals and married career women revealed significant correlations between role ambiguity and role expectancy with reverse predictability. Nonetheless, the relationship between role ambiguity and role expectancy of university educators has not yet been examined. However, many scholars (Bergström, 2019; Goldhaber et al., 2019; Kempe and Grönlund, 2019) indicated that teachers play a crucial role in curriculum implementation and development, and therefore, they need to be aware of their identity in it. As described in the research background of this study, teachers’ professional roles change over time, and that is why different interpretations follow one another. Regarding teachers’ role in curriculum development, many scholars (Hershkovitz and Karni, 2018; Prince, 2018; Doron and Spektor-Levy, 2019) believed that it should be limited to a passive position, such as approving and implementing courses. Given these views, we should focus on the professional role of teachers. Better learning outcomes are usually the result of a better practice of professional roles. When developing a curriculum, the extent to which teachers practice their roles is critical to implementation results. The importance of teachers’ role practice can also be understood in this context. Therefore, this study investigates the role ambiguity, role expectation, and practice of university faulty in business administration departments. It is expected that the results of the study will help national university faculty to show professional spirits and attitudes in their professional activities, which will greatly contribute to the promotion of educational standards in national universities.

## Literature Review and Hypotheses

Zahwa et al. (2020) conducted a qualitative questionnaire survey to investigate the practices and affective state of the university faculty in the field of science and technology and found that most university faculties were familiar with course content and instructional design. However, they did not understand the use of the technology and considered instructional videos to be an important tool better suited for distance education. Espino-Díaz et al. (2020) noted that distance education was practiced to cope with COVID-19; yet, even teachers and students in advanced countries experienced a lack of the necessary equipment for distance education or fast bandwidth. The questionnaire survey of students revealed that a high proportion of problems were caused by the Internet speed and equipment in residential areas. Departmental responses and faculty interviews also indicated that faculty had the anxiety of mastering distance education technologies at the beginning of distance education (Yen et al., 2018). To cope with distance education practiced due to COVID-19, different pedagogical methods should be adapted in the process. Nevertheless, it was a challenge for teachers who were forced to suddenly switch from face-to-face to distance education. Although most teachers felt that they performed well in distance education, 16.7% of the students stated that teachers needed to improve themselves in terms of the distance education pedagogy (Kamali, 2021). Teachers might consider the ability to use synchronous video tools or recorded lessons as a satisfactory way of distance education; in reality, this is far from high-quality distance education. Moreover, students’ learning effectiveness needs to be better tested as well (Yen et al., 2018).

Maden-Eyiusta (2019) explained role ambiguity as the lack of clear information about role expectation, how to fulfill role expectation and role performance. Clemen et al. (2018) indicated that several researchers mentioned indefinite or insufficient information about the individual role when defining role ambiguity. In this situation, people could not clearly define the work goals and responsibilities, which results in negative effects and pressure and causes ambiguous role expectations. Yester (2019) explained role expectation as the individual or group expectation of a certain role, which includes an individual expectation of a certain role and the groups’ or others’ expectation of the role. If an individual perceives the role differently than the needs or expectations of others due to role ambiguity, this results in role expectation differences. Hughes and Evans (2018) considered role expectation as the expectation and requirement of role takers and role givers. If a role taker exhibits role ambiguity that causes a difference in the role giver’s role expectation. This will lead to role expectation differences. Accordingly, this study hypothesizes that **H1:** role ambiguity presents negative and significant effects on role expectation.

In a study of military instructors, Smith and Sweet (2019) found out the significant effects of role behavior and role practice. In this study, the role behavior model showed role congruence with others’ expectations and self-expectation. Martínez-Díaz et al. (2020) pointed out that role expectation is the public expectation of the behavior of a person with a specific social status. This also includes self-expectation and public expectation of the role. Al-Nasser and Al-Enezy (2018) concluded in their study that military instructors’ role behavior and role practice influence each other. Military instructors who were reinforced in their role behavior performance improved their job performance. When role behaviors are consistent with others’ and their own expectations, they receive positive evaluations and demonstrate better role practice. In a study of higher education professionals, Kanellakis et al. (2018) found that the factors of role practice included role expectation, workload, and professional knowledge or competence. In this study, role expectation was found to have positive correlations with role practice. Accordingly, higher education professionals with high personal role expectations showed better role practice. Accordingly, this study hypothesized that **H2**: role expectation has positive and significant effects on role practice.

Yan et al. (2021) considered role practice as a person’s actual performance during role tasks or behaviors. Zhang and Sun (2020) referred role practice as the actual behavior of a role performed in the society related to the role. Zambrana et al. (2019) study revealed significant negative correlations between role ambiguity and role practice, and they concluded their study by suggesting that a bank that emphasizes role conflict and role ambiguity of financial advisors may contribute to the job satisfaction of financial advisors, which in turn improves their role practice. Bloomingdale and Darmody (2019) discovered that there were more senior financial advisors than junior financial advisors among the study participants and suggested providing employees with clearer direction and work objectives to better understand the expectations of the bank and individual responsibilities to reduce role ambiguity and promote role practice. It was necessary to reduce role ambiguity and increase self-efficacy to promote role practice among managers. Mitchell et al. (2018) mentioned in their study that directors with uncertain and ambiguous work contents may not be aware of job objectives, scope, program, and responsibilities and tasks. They concluded in their study that research participants’ role ambiguity was caused by the unfamiliarity with company regulations and objectives. Therefore, the participants of the study were suggested to fully understand corporate regulations, operational objectives, and applicable resources and programs. Consequently, this study hypothesizes that **H3**: role ambiguity has significant negative effects on role practice.

**FIGURE 1:**
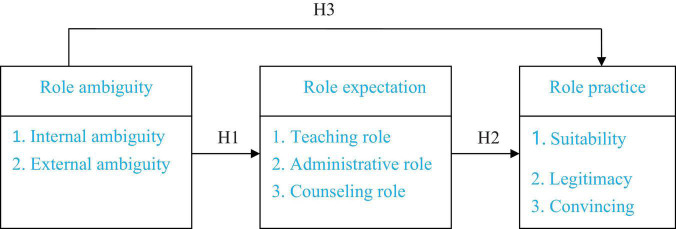
Conceptual structure.

Based on the above literature, it is considered in this study that there is a relationship among role ambiguity, role expectation, and role practice. The influence paths and the hypothesis tests conducted in this study are illustrated in Figure 1.

## Methodology

### Operational Definition

#### Role Ambiguity

With reference to Lin and Ling (2018), the role ambiguity in this study can be defined with two dimensions.

1. Internal ambiguity: the absence of information about role definition, expectations, job title, behavior, and ethics when interacting with the internal organization.
2. External ambiguity: the absence of information about role definition, expectations, responsibilities, tasks, behaviors, and ethics when interacting with external members of the organization.

#### Role Expectation

Following Shin and Gonzalez (2018), teachers’ role expectation was defined with three dimensions in this study.

1. Teaching role includes instructional objectives, curriculum design, teaching skills, environment design, instructional resources, and instructional assessment.
2. Administrative role covers administrative work and interaction with parents and other people, as well as teachers’ self-improvement.
3. Counseling role includes respecting, understanding, and guiding students, as well as conducting a healthy teacher–student interaction.

#### Role Practice

Based on Shea et al. (2019) study, three key dimensions were considered for the success of role practice.

1. Suitability: the suitability of the behavior performed for the position, conformity to situations, and the correctness of the practice behavior.
2. Legitimacy: whether the performed behavior conforms to regulations and is good behavior.
3. Convincing: whether the performed behavior convinces others and the practice is sincere.

### Research Method

A questionnaire survey refers to a standard questionnaire designed by the researcher for the respondent. Questionnaires are used to collect current facts, improve current standards, draft the topic of the plan, decide measures, and provide research references (Eden and Ackermann, 2018). Schwab-McCoy (2019) pointed out the following advantages of a questionnaire survey. (1) The copy and the postage costs for the questionnaire are low and it is easy to implement. (2) Respondents can answer freely without the psychological burden of interacting directly with the researcher. (3) The content of the items is consistent, so that they can be easily compared and standardized. In a questionnaire survey, the researcher can prepare a questionnaire with the studied items and send it to the respondents to collect the data. Respondents are selected in representative samples for the study to determine the mutual effects, distribution, and reciprocal relationships among variables (Kwiek, 2020). Considering the research objectives, research problems, and the appropriateness of different research methods, a questionnaire form was used in the survey research of this study.

### Questionnaire Design

The questionnaire was designed based on the relevant literature. The draft questionnaire was formed after in-depth interviews with the experts. After the pretest, the preliminary questionnaire’s reliability was calculated for the formal questionnaire. In the development process of the preliminary questionnaire, expert interviews were conducted on the validity of the questionnaire, and expert opinions were received for the content validity of the questionnaire. The expert review refers to the in-depth interviews with six scholars and six senior teachers to receive their opinions for the questionnaire items. The preliminary questionnaire was revised according to the experts’ opinions. Faculty members from six universities were invited to fill in the preliminary questionnaire. Then, 1 week after sending the questionnaire, the responses of the ones who did not return the questionnaire were collected with phone calls to increase the response rate. It was aimed to retrieve 50 copies of questionnaires for the analysis.

### Participants of the Study

The participants of this study were composed of faculty members in business administration departments of universities in China. In the process of data collection, 450 copies of questionnaires were distributed. A total of 363 valid copies were retrieved, with a retrieval rate of 81%.

### Reliability and Validity Tests

Confirmatory factor analysis (CFA) is an important component of SEM. For this reason, the measurement model should be tested with a two-stage model modification during CFA before the structural model evaluation. When the model fit is acceptable, a second-step SEM is conducted. The factor loadings of the dimensions in the model were found as between 0.60 and 0.80. The composite reliability was found between 0.75 and 0.90, and the average variance extracted was between 0.60 and 0.80. These results meet the standards of (1) factor loading > 0.5, (2) composite reliability > 0.6, and (3) average variance extracted > 0.5. Thus, the dimensions show convergent validity.

## Results

### Structural Model Analysis

The structural model analysis includes the analysis of model fit and explanatory power of the overall research model. According to the researchers’ opinions, seven numerical indices were used for testing the overall model fit. These tests included the chi-square (χ2) test, the χ2-degree of freedom ratio, the goodness-of-fit index, the adjusted goodness-of-fit index, the comparative fit index, the comparative hypothesis model, and the chi-square difference of independent model.

To sum up, the χ2-degree of freedom ratio is used to test model fit and it is better when smaller. The χ2-degree of freedom ratio of this research model was found as <3 (1.36). GFI and AGFI are better when they are close to 1, without an absolute standard for the fit. Accordingly, GFI > 0.9 and AGFI > 0.8 can be considered as acceptable. GFI and AGFI in this study were found as 0.95 and 0.86, respectively. RMSEA between 0.05 and 0.08 reveals a good model with a reasonable fit; RMSEA in this study was calculated as 0.07. The acceptable standard of CFI is >0.9; CFI in this study was revealed as 0.92. NFI should be higher than 0.9; NFI in this study was found as 0.90. Overall speaking, the goodness-of-fit indices conform to the standards, which reveals an acceptable model of the research results. Therefore, the research patterns can be used to explain the formal research data.

From the above overall model fit indices, it can be seen that the model structured in this study and the observed data have favorable goodness of fit, which means that the theoretical model can fully explain the observed data. After testing the model fit, the correlation coefficients and coefficient estimates between role ambiguity, role expectation, and role practice can be better understood.

The research data were organized in Table 1. The overall results of the model analysis showed that two factors in role ambiguity (internal ambiguity and external ambiguity) can significantly explain role ambiguity (t > 1.96, *p* < 0.05). Similarly, three factors in role expectation (teaching role, administrative role, and counseling role) can significantly explain role expectation (t > 1.96, *p* < 0.05). Finally, three factors in role practice (suitability, legitimacy, and convincing) can significantly explain role practice (t > 1.96, *p* < 0.05). Obviously, the overall model presents a good preliminary fit.

**TABLE 1:** Overall results of linear structural model analysis.

Evaluation item	Parameter/evaluation standard		Result
Preliminary fit	Role ambiguity	Internal ambiguity	0.65*
		External ambiguity	0.67*
	Role expectation	Teaching role	0.74**
		Administrative role	0.73**
		Counseling role	0.71*
	Role practice	Suitability	0.66*
		Legitimacy	0.75**
		Convincing	0.77**
Internal fit	Role ambiguity→role expectation		−0.83***
	Role expectation→role practice		0.85***
	Role ambiguity→role practice		−0.88***

*p < 0.05, **p < 0.01, ***p < 0.001.

In terms of the internal fit, the role ambiguity showed negative and significant correlations with role expectation (−0.83, *p* < 0.01), role expectation revealed positive and significant correlations with role practice (0.85, *p* < 0.01), and role ambiguity was found to be negative and revealed statistically significant correlations with role practice (−0.88, *p* < 0.01). According to the results, H1, H2, and H3 were supported.

## Discussion

The work content of university faculty in business administration has changed with the needs in different places. So, school supervisors can have different requirements for teachers. In addition, school supervisors do not have sufficient knowledge about university faculty in business administration. As a result, high expectations are placed on university faculty in business administration and they are even assigned other administrative tasks, which leads to an additional burden and even disrupts the rhythm of teaching. This situation results in role ambiguity on university faculty in Business Administration. As a result, they have to constantly communicate with their supervisors about the difference between their own expectations and the supervisors’ expectations, which leads to delays in teaching. To cope with the global rage of the COVID-19 pandemic, many schools have implemented “distance learning” to avoid interrupting students’ schoolwork (Chew et al., 2020). COVID-19 pandemic has changed the world, people’s lives, and their learning styles, which accelerates the development of distance education (Garfin et al., 2020). Faced with unfamiliar teaching styles, teachers show anxiety and confusion (Kamali, 2021). In such a chaotic period, the application of distance education to students causes teachers to experience difficulties in evaluating each student’s learning situations (Lupe et al., 2020), and the difficulty of maintaining order in the classroom increases to increase teachers’ role pressure in teaching activities (Lai et al., 2020). It is suggested that school supervisors clarify and improve the knowledge of university faculty in business administration. They should also help the teachers to understand the work content and limits through mutual communication and encouragement, support the teaching work, and have university faculty in business administration focus on teaching activities to enhance the role practice at work. University faculty in business administration, on the other hand, need to increase their professional knowledge to enhance their professional status and be interested in and capable of conducting research on education. Professional development is a continuous process. To adapt to the social changes and the development of the world trend, the continuous professional development of university faculty in business administration is an important way to improve their professional knowledge. Accordingly, university faculty in business administration who conduct more studies on education and publish more research results and experiences on journals to manage the professional image can help the public recognize their professional image.

## Conclusion

The research results revealed that the regulations for the work content of university faculty in business administration and various systems are not fully planned. Therefore, university faculty in business administration feel the need to clarify various regulations, responsibilities, work content, and work objectives to improve their devotion to work and achieve a higher level of ability. University faculty in business administration who are willing to engage in teaching activities are ambitious, have high expectations of themselves and their work, and strive to support students in the learning process. University faculty in business administration has a high expectation of the provision of student and parent counseling, evaluation of problems, promotion of teaching, and the integration of surrounding resources. With high role expectations, the role practice to display the ability to work and the promotion of teaching can be satisfactory. The research results revealed that university faculty in business administration have high role expectations during low role ambiguity which facilitates role practices. In view of the ever-changing educational environment, university faculty in business management need to constantly pursue new knowledge, improve teaching, enrich the content, participate in study- or education-related workshops, and enhance their teaching and counseling abilities to reduce role ambiguity and achieve role expectation and role practice (Ho et al., 2021). The administration unit should organize workshops according to the teachers who need to reduce the fear of negative reactions (Tabancali and Su, 2021). Teachers should be encouraged to explore new topics and new ways of thinking and apply the acquired knowledge in the classroom to promote students’ learning ability and gain parents’ trust. Complementarity among different stakeholders can reduce role ambiguity (Lin and Chuang, 2018).

## Data Availability Statement

The original contributions presented in the study are included in the article/supplementary material, further inquiries can be directed to the corresponding author.

## Ethics Statement

This study was conducted in accordance with the recommendations of the Ethics Committee of the Fujian Business University, and written informed consent was obtained from all the participants. All participants were asked to read and approved the ethical consent form before participating in this study. The participants were also asked to adhere to the guidelines in the form during the implementation. The research protocol was approved by the Ethical Committee of the Fujian Business University.

## Author Contributions

YD performed the initial analyses and wrote the manuscript. HZ, AX, and YC assisted in the data collection and data analysis. All authors revised and approved the submitted version of the manuscript.

## Conflict of Interest

The authors declare that the research was conducted in the absence of any commercial or financial relationships that could be construed as a potential conflict of interest.protect

## Publisher’s Note

All claims expressed in this article are solely those of the authors and do not necessarily represent those of their affiliated organizations, or those of the publisher, the editors and the reviewers. Any product that may be evaluated in this article, or claim that may be made by its manufacturer, is not guaranteed or endorsed by the publisher.
